# Identification of effective spreaders in contact networks using dynamical influence

**DOI:** 10.1007/s41109-021-00351-0

**Published:** 2021-01-19

**Authors:** Ruaridh A. Clark, Malcolm Macdonald

**Affiliations:** Department of Electronic and Electrical Engineering, University of Strathclyde, George Street, Glasgow, UK

**Keywords:** Disease spread, Dynamical influence, Network structure

## Abstract

Contact networks provide insights on disease spread due to the duration of close proximity interactions. For systems governed by consensus dynamics, network structure is key to optimising the spread of information. For disease spread over contact networks, the structure would be expected to be similarly influential. However, metrics that are essentially agnostic to the network’s structure, such as weighted degree (strength) centrality and its variants, perform near-optimally in selecting effective spreaders. These degree-based metrics outperform eigenvector centrality, despite disease spread over a network being a random walk process. This paper improves eigenvector-based spreader selection by introducing the non-linear relationship between contact time and the probability of disease transmission into the assessment of network dynamics. This approximation of disease spread dynamics is achieved by altering the Laplacian matrix, which in turn highlights why nodes with a high degree are such influential disease spreaders. From this approach, a trichotomy emerges on the definition of an effective spreader where, for susceptible-infected simulations, eigenvector-based selections can either optimise the initial rate of infection, the average rate of infection, or produce the fastest time to full infection of the network. Simulated and real-world human contact networks are examined, with insights also drawn on the effective adaptation of ant colony contact networks to reduce pathogen spread and protect the queen ant.

## Introduction

Despite the commonality of spreading process—such as consensus, disease spread, and rumour propagation—De Arruda et al. (2014) notes that the efficacy of centrality measures, in identifying effective spreaders, differs depending on the system dynamics. Given the clear relationship between random walk and spreading processes it is notable that the efficacy of eigenvector-based spreader selection also varies with the system dynamics; Clark et al. (2019) details the efficacy of eigenvector assessment for consensus dynamics, while De Arruda et al. (2014) notes the inferiority of eigenvector centrality for determining the influence of disease spreaders. In this work we introduce a new network representation of disease spread dynamics, to demonstrate that eigenvector-based assessments can be effective across differing spreading processes as long as the network accurately captures the system dynamics.

For disease spread dynamics, degree-based metrics (which include k-shell/k-core strategies) have been repeatedly found to identify a system’s effective spreaders as in De Arruda et al. (2014), Kitsak et al. (2010), Da Silva et al. (2012), Zeng and Zhang (2013), Wang et al. (2016), Liu et al. (2016), Salamanos et al. (2017) and Jiang et al. (2019). While, k-shell and s-core [see Eidsaa and Almaas (2013)] strategies make some acknowledgment of the network structure, these methods are largely agnostic to the communities that may exist even if these are highly separated from each other. In consensus dynamics, Clark et al. (2019) note that the most effective strategies for spreading information often relies on distributing spreaders across multiple communities rather than locating them centrally in the network. Liu and Hu (2005) and Stegehuis et al. (2016) found that community structure does influence the spread of disease on networks. Therefore we aim to highlight that, as with consensus dynamics, a system’s eigenvectors capture the interplay of dynamics and network structure that is fundamental to determining the effectiveness of disease spreaders.

Network structure, without considering the system dynamics, has been incorporated into the detection of effective spreaders, with Ghalmane et al. (2019) expanding on the concept of modular centrality to improve the performance of common centrality measures. Degree-based metrics have also been developed that only decentralise the spreader location rather than explicitly acknowledge the network’s structure, as in Kitsak et al. (2010), Zeng and Zhang (2013), Wang et al. (2016), Jiang et al. (2019) and Yang et al. (2019). These include selecting spreaders from less prominent hubs [see Jiang et al. (2019)], preventing neighbouring spreaders from being selected [see Kitsak et al. (2010) and Wang et al. (2016)], altering the selected spreader’s degree to be negative to avoid selecting nodes with overlapping spheres of influence [see Yang et al. (2019)], and acknowledging that selecting a spreader should diminish the importance of the links to an already infected node [see Zeng and Zhang (2013)]. These methods are agnostic to the interplay of topology and network dynamics, which can lead to inaccuracies for certain topologies, as acknowledged by Namtirtha et al. (2020) where a tunable optimisation is developed. In contrast we ensure that every connection informs the spreader selection with the system’s eigenvectors providing a holistic assessment of the network, as demonstrated in Clark et al. (2019) and Punzo et al. (2016) for networks with linear consensus dynamics.

The networks considered herein are contact networks, constructed from close proximity contact durations and frequently used to analyse how disease can spread through a group of individuals as in Vanhems et al. (2013), Salathé et al. (2010), Stehlé et al. (2011), Génois et al. (2015) and Génois and Barrat (2018). These networks are representative of human interactions where human-to-human disease spread can occur. Information on the order of interactions is lost by treating the system as a static network, but these networks are still useful for understanding likely pathways for disease progression. However, as discussed, a new approach for representing these contact networks is introduced in this work to capture the non-linear relationship between contact duration and the probability of disease spread. This relationship is often described with an exponential function, as in Kiss et al. (2017) where it is used to simulate disease spread on networks. In this paper, we shall explore how to account for this exponential relationship when using eigenvectors to detect effective spreaders, and as a consequence highlight the important role contact network structure can play in disease spread.

## Methods

### Network definition

A graph is defined as $G=(V,E)$, where there is a set of $V$ vertices and $E$ edges, which are unordered pairs of elements of $V$ for an undirected graph and ordered pairs in a directed graph. The adjacency matrix, *A*, is a square $\text {N} \times \text {N}$ matrix when representing a graph of $\text {N}$ vertices. This matrix captures the network’s connections where $(A)_{ij}=(A)_{ji}>0$ if there exists an edge connecting vertex *i* and *j* and 0 otherwise. This paper only concerns the detection of effective spreaders, therefore all contact networks are scaled to be within the same range, $0\le (A)_{ij}\le 1 ~\forall \, i,j \in V$. Variable edge weights contain information on the relative strength of interactions, which for contact networks is defined by contact duration. The adjacency matrices throughout this paper are symmetric, but it will be important to note that the nonzero row entries of *A* indicate outgoing links from which a node can contract disease. Whereas the nonzero column entries are incoming links that a node can use to spread disease to its contacts.

### Communities of dynamical influence

The communities of dynamical influence (CDI), introduced by Clark et al. (2019), are used to provide evidence of the prominent role network structure can play in determining a node’s influence as a spreader of disease. This community detection attempts to capture the dynamical influence of nodes, where Klemm et al. (2012) defines dynamical influence as the influence a node has over the dynamic state of other nodes in the network. CDI are defined by global and local dynamical influence, with these communities identified by embedding the network in a Euclidean space defined by the real part of the system’s three most dominant eigenvectors. Community leaders are identified as nodes that are further from the origin of this coordinate frame than their neighbours. The nodes belonging to each leader’s community must have a directed path connecting them to the leader. If multiple leaders are viable then assignment is based on alignment to a leader, assessed using the scalar projection of each node’s position vector in Euclidean space with respect to the viable leaders. In terms of dynamical influence, with respect to the whole network (i.e. global), this approach produces the same results as eigenvector centrality. However, Clark et al. (2019) show that more localised influence can also be identified by incorporating the 2nd and 3rd dominant eigenvectors, with community leaders not necessarily prominent according to eigenvector centrality.

### Susceptible-infected (SI) simulations

The main focus of this paper is the effectiveness of disease spreaders on contact networks. The performance of spreaders is analysed using susceptible-infected (SI) simulations, see Miller and Ting (2020), where edge weight affects the probability of transmission, with the time to infection exponentially distributed as described by Kiss et al. (2017). This paper is primarily concerned with the influence of a node on the spread of disease, therefore susceptible-infected-susceptible (SIS) and susceptible-infected-recovered (SIR) models are not considered to reduce the effect of stochastic resusceptibility and recovery events. SI simulations guarantee that all nodes with a directed path to an infected node will eventually become infected, therefore the effectiveness of disease spreaders are monitored by tracking the time taken for 25%, 50%, 75%, and 100% of the network to become infected.

### Exponentially Distributed Contact Networks

For simulating the spread of disease through a network, exponentially distributed times to infection [as in Miller and Ting (2020)] are commonly assumed but contact network analysis frequently uses contact times to weight the adjacency matrix, such as in Salathé et al. (2010). In this work, the adjacency matrix is altered to reflect the exponential relationship between contact time and risk of disease spread, with the aim of improving the identification of effective spreaders of disease. The first step is to apply a commonly used exponential function for converting contact time into a weight that more accurately represents the increased risk of transmission due to the passage of time,

1 $$\begin{aligned} P = 1-exp(-\tau t) \end{aligned}$$

where *t* is a contact time between two nodes in the network and $\tau$ is the transmission rate, see Kiss et al. (2017). By scaling each contact time according to Eq. 1, an exponentially adjusted adjacency matrix, $A_e$ is produced where

2 $$\begin{aligned} (A_e)_{ij} := {\left\{ \begin{array}{ll} 1-exp(-\tau (A)_{ij}), &{} \hbox {if}\ (A)_{ij}>0.\\ 0, &{} \text {otherwise}. \end{array}\right. } \end{aligned}$$

Therefore, $A_e$ is more representative of the probability that an infection will travel over an edge than *A*.

When multiple infected nodes are connected to a susceptible node, the system dynamics are no longer accurately captured by the adjusted adjacency. For example, when two infected nodes are in contact with the same susceptible node the risk of transmission is less than the sum of the two edge weights (even when using $A_e$). Instead, the risk of transmission is more accurately captured by applying Eq. 1 when *t* is equal to the sum of the contact times with both of the infected nodes. Therefore, an exponentially adjusted Laplacian matrix, $L_e$, is also proposed where the diagonal elements are equal to *P* in Eq. 1 when $t=\sum _j (A)_{ij}$, i.e. the sum of all contact times for a given node. The off-diagonal element are composed of the, negated, adjusted adjacency matrix values, i.e.

3 $$\begin{aligned} L_e = D_{e_{\Sigma t}} - A_e \end{aligned}$$

where $$(D_{e_{\Sigma t}})_{ii}=diag(1-exp(-\sum _m (A)_{im}))$$. Hence,

4 $$\begin{aligned} (L_e)_{ij} := {\left\{ \begin{array}{ll} -(1-exp(-\tau (A)_{ij})), &{} \hbox {if}\ (A)_{ij}>0.\\ 1-exp(-\sum _m (A)_{im}), &{} \text {if i = j} \\ 0, &{} \text {otherwise}. \end{array}\right. } \end{aligned}$$

### Understanding influence in disease spread

The assessment of influence in directed networks, following linear consensus, can be captured by the first left eigenvector $\text {v}_1$ as described by Clark et al. (2019). The ratio of indegree to outdegree affects $\text {v}_1$, where nodes with a high indegree and low outdegree wield more influence since their state has a greater impact on their neighbours than their neighbours’ states have on them, see Clark et al. (2019). This is not usually relevant for undirected networks but, as discussed, the exponentially adjusted Laplacian $L_e$ creates an imbalance between incoming connections (along which it can spread disease) and the diagonal entry—that usually is the sum of outgoing connections—but now equals the exponentially adjusted sum of contact times. The ratio between incoming edge weights and the magnitude of a node’s diagonal entry is greatest for nodes with a high degree. This results in high degree nodes being rewarded with an amplification of their influence, as determined by $\text {v}_1$, when compared with a linear system. Influence in this context means that a node is effective at spreading the disease to others, while not being easily infected itself by a more influential node. Both aspects, the ability to spread and the difficulty to infect, will be important in the following sections where we will describe how to optimise $L_e$ to select effective spreaders.

### Disease spreader selection

A challenge in this work is translating approaches from linear consensus to disease spread. Finite resources were allocated to nodes in Clark et al. (2019) to drive any network to rapid consensus. Resource were allocated with an optimisation based on CDI’s community designations and the first left eigenvector ($\text {v}_1$). Such an approach needs to be adapted when considering disease spread; nodes have susceptible and infected states that are binary unlike the variable resource allocations and smooth transitions seen in consensus models. The first left eigenvector $\text {v}_1$ is still the basis of spreader selection presented herein, but to select any number of disease spreaders an iterative process of spreader selection and network alteration is introduced. For an undirected Laplacian matrix *L* ($L=D-A$ where *D* is a diagonal matrix of degree), $\text {v}_1$ (where $\lambda _1=0$) is a uniform vector. The exponentially adjusted Laplacian $L_e$ is still undirected but the imbalance between incoming weight sum and the magnitude of the diagonal entry means that $\text {v}_1$ of $L_e$ is no longer uniform and can be used to select influential spreaders. Also $\lambda _1 \ne 0$, but $\lambda _1$ is still the smallest eigenvalue of $L_e$.

Spreader selection is an iterative process, where the node associated with the largest entry of $|v_1|$ is the first selected. If more spreaders are to be selected, then the *i*-th selected node $\gamma [i]$ has its incoming and outgoing connections removed, i.e. $(L_e)_{\gamma [i]j}=(L_e)_{j\gamma [i]}=0$ $\forall \,j \ne \gamma [i] \in {\mathcal {V}}$, and $(L_e)_{\gamma [i]\gamma [i]}=\sum _j (L_e)_{jj}$. A new $\text {v}_1$ is calculated for this updated $L_e$ matrix and the process repeats until all spreaders are selected. By setting $(L_e)_{\gamma [i]\gamma [i]}=\sum _j (L_e)_{jj}$, this ensures that the smallest eigenvalue of $L_e$ is not associated with an already chosen spreader node but instead concerns the giant component of the graph. The spreader selection algorithm is detailed in pseudo-code in Algorithm 1. 
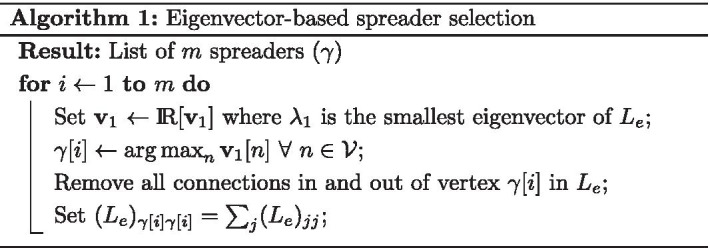


### Optimising spreader selection

The exponentially adjusted Laplacian $L_e$ is only an approximation of disease spread dynamics. This section details how understanding the implications of that approximation enables different types of effective spreader to be identified. Spreaders can provide a fast initial rate of infection, a fast time to full infection of the network, or a compromise between those two that provides a more consistent rate of infection.

Spectral analysis, in the form of the first left eigenvector, uses $L_e$ to compare a node’s ability to spread infection, when all its neighbours are susceptible, with the ability of its neighbours, if they were all infected, to spread disease to it. This is obviously a comparison of two extremes, which as discussed previously provides greater reward to nodes with a high degree. The matrix $L_e$ represents the maximum reward, in terms of influence, that could be expected from the non-linear relationship between contact duration and the probability of disease spread. By scaling the contact durations so that they are reduced in the adjacency matrix *A*, before creating $L_e$, the ratio of incoming connection weights (ability to spread disease) to the magnitude of the diagonal entry of $L_e$ (ability to catch disease) is reduced. Hence, the reward in terms of influence for high degree nodes can also be reduced through scaling the adjacency matrix.

Scaling the adjacency matrix is achieved by adjusting $\text {s}$, where the exponentially adjusted adjacency becomes

5 $$\begin{aligned} (A_e)_{ij} := {\left\{ \begin{array}{ll} 1-exp(- {\tau }{s} (A)_{ij}), &{} \hbox {if}\ (A)_{ij}>0.\\ 0, &{} \text {otherwise}. \end{array}\right. } \end{aligned}$$

The exponentially adjusted Laplacian becomes

6 $$\begin{aligned} (L_e)_{ij} := {\left\{ \begin{array}{ll} -(1-exp(-{\tau }{s}(A)_{ij})), &{} \hbox {if}\ (A)_{ij}>0.\\ 1-exp(-\sum _m {\tau }{s}(A)_{im}), &{} \text {if i = j} \\ 0, &{} \text {otherwise}. \end{array}\right. } \end{aligned}$$

It is anticipated that s values close to 1, which reward high degree nodes, will be most effective for detecting spreader selections that maximise the initial rate of spread. Conversely, low s values are expected to result in selections that identify nodes belonging to more isolated communities in the network and hence provide better results when looking to infect the whole network. However, the best value of s varies depending on the network so an optimisation is introduced where a set of discrete values between 1 and 0.001, $S=\{0.001,0.1,0.2,0.3,0.4,0.5,0.6,0.7,0.8,0.9,1\}$, are tested where $s \in S$. For each value of s, a spreader selection is generated and all unique selections tested with SI simulations described in Miller and Ting (2020). While the set of *S* provides effective selections for most of the topologies tested, the results shall highlight certain artificial topologies for which a smaller range of values, such as $S=\{0.001,0.01,0.02,0.03,0.04,0.05,0.06,0.07,0.08,0.09,0.1\}$, produces superior selections.

#### *Initial*—maximising initial spread

The *Initial* selection aims to maximise the initial rate of infection spread, by comparing the time to 25% infected for each of the candidate spreader selections. The chosen selection produces the minimum median time from 10 SI simulations.

#### *Averaged*—consistent performance

Times are recorded at 25%, 50%, 75%, and 100% intervals of network infection from 10 SI simulations. For a given candidate spreader selection, the *Averaged* selection records the median times produced at each percentage interval. The median times are then divided by the mean of these medians, producing a normalised median time at each percentage interval, with the sum of these normalised medians assessed. The selection that minimises the sum of normalised median times is selected.

#### *End*—fast time to full infection

The *End* selection minimises the time to 100% infected, by comparing each of the candidate spreader selections and selecting the option that produces the minimum mean time from 10 SI simulations.

#### Weighted degree (no neighbours)

The benchmark for performance presented in the paper is weighted degree (no neighbours), which is shown by Kitsak et al. (2010) to be highly effective at spreader selection. This method combines weighted degree-based selection with the restriction that neighbours of spreaders already selected are not viable for inclusion, referred to here as weighted degree (no neighbours). This approach is an acknowledgment that weighted degree centrality, and also k-shell strategy [see Kitsak et al. (2010)], are largely ignorant to the network structure and susceptible to error when weakly connected communities are present. However, the effectiveness of the no neighbour restriction only confirms that a node becomes less influential, in terms of disease spread, when a neighbour becomes infected. This reduction in influence could be expected as an infected node has no influence over a node that is already infected.

Other benchmarks were used—including eigenvector centrality, s-core strategy (a weighted degree version of k-core/k-shell), and betweenness centrality—and the results from these approaches are included in Additional file 1: Fig. S1 and S2.

## Results

### Visualising spreader selection

**Fig. 1:**
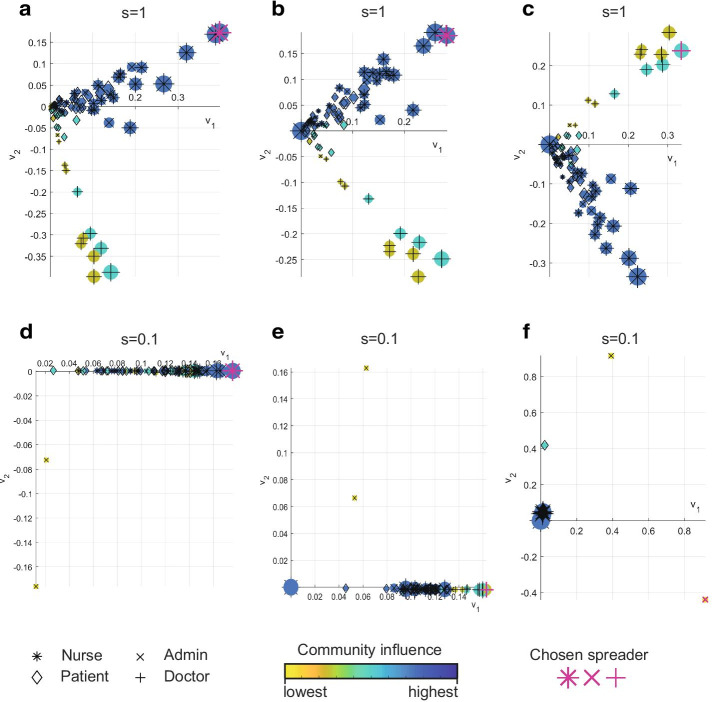
Spreader selection for hospital ward contact network ($\tau = 1$). CDI determined communities, defined for **a** are highlighted in **b**–**f** to visualise the iterative process of spreader selection when $s=1$ (**a**–**c**) and $s=0.1$ (**d**–**f**). In **a**, **d** the original network is embedded in a Euclidean space defined by the dominant eigenvectors ($\text {v}_{1}$ and $\text {v}_{2}$) of $L_e$. The node with the largest $\text {v}_{1}$ value is the chosen spreader. In **b**, **e**, $L_e$ is updated by removing connections from the chosen spreader. In **c**, **f** $L_e$ is updated again by removing connections from the chosen spreader in **b**, **e** respectively. Markers denote hospital role and dot colour denotes community where community influence is ranked in **a** according to largest $\text {v}_{1}$ entry in each community. Node size is mostly proportional to weighted degree, with a minimum size limit used to aid visibility

Algorithm 1 is shown in operation in Fig. 1 for $s=1$ (in Fig. 1a, b, c) and $s=0.1$ (in Fig. 1d, e, f), where it is applied to a hospital ward contact network from Vanhems et al. (2013) to identify three spreaders. Fig. 1 presents a Euclidean space defined by the first two left eigenvectors of the exponentially adjusted Laplacian matrix ($\text {v}_1$ and $\text {v}_2$). In Fig. 1a the most prominent node according to $\text {v}_{1}$ is the first chosen spreader from the network defined using $s=1$. The communities of dynamical influence (CDI), described in the Communities of dynamical influence section, divides the network into communities with their influence dependent on the largest $\text {v}_{1}$ entry. Fig. 1b details how removal of this chosen spreader, from the influential nurse-dominated community, results in other nodes in that community losing influence (i.e. a smaller $\text {v}_1$ value) as the number of nodes and pathways for spreading disease in that community has decreased. When a second node from the same community is removed, Fig. 1c details further loss of influence for the nurse community with the chosen spreader now located in a doctor-dominated community.

When $s=0.1$ the process of spreader selection follows the same pattern, but as can be seen already in Fig. 1d the least influential community from Fig. 1a is given more prominence. In fact, after removing two chosen spreaders, the most prominent node in Fig. 1f is a node from this least influential community (where communities are kept the same as defined for the network in Fig. 1a). Importantly, and unlike degree based metrics, after selecting a spreader the next spreader is chosen based on a holistic assessment of node influence, i.e. an updated $\text {v}_1$ for the updated network topology after chosen spreader removal. In this way, every spreader selection in this eigenvector-based selection is dependent on and responsive to network structure.

When $s=1$, two nurses are selected in Fig. 1a, b from the same community with the high weighted degree nodes ensuring rapid initial spread of disease. In Fig. 1c, a doctor is selected as the next most effective spreader as there is a clear separation between the doctor and nurse dominated communities. In contrast, when $s=0.1$, for Fig. 1e–f the system dynamics give more prominence to isolated nodes and communities that are difficult to infect. In Fig. 1d the chosen spreader is the same prominent nurse as selected in Fig. 1a. However, in Fig. 1e a doctor from the less influential doctor community is chosen, rather than another nurse. Finally, in Fig. 1f an isolated member of admin staff is selected since they are hard to infect due to their limited contact with others in the network.

### Community spread

**Fig. 2:**
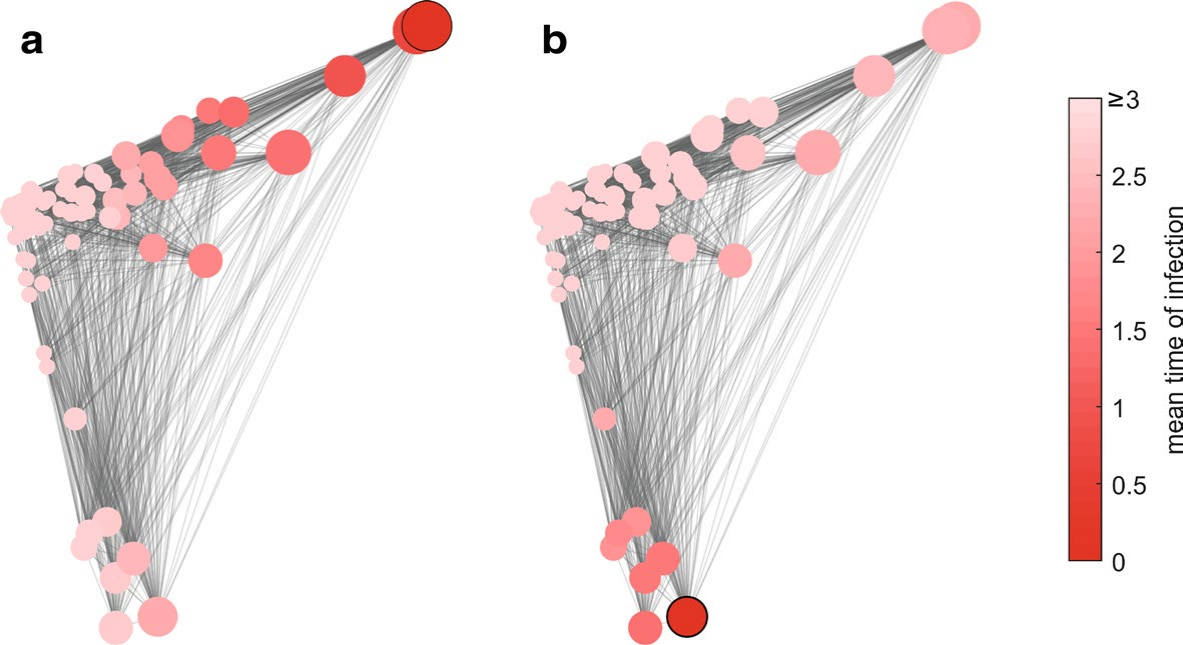
Simulated initial spread of disease from key nodes in a hospital ward contact network. A black outline defines the initially infected node in **a**, **b**. The mean time to infection, from 100 SI simulations ($\tau = 1$), is differentiated by colour for values below 3. Connections between nodes are also displayed

The eigenvector-based selection, visualised in Fig. 1, selects spreaders from different communities. Fig. 2 reveals why this is an effective strategy, as the initial spread of disease from prominent community nodes is primarily within their own community. The choice of $\tau = 1$ is not intended to be representative of a particular disease, therefore time is reported without units and used only for comparison throughout this section. In Fig. 2a, disease is spread from the most prominent nurse in the nurse-dominated community, highlighted in Fig. 1a. The smallest mean times to infection are within the nurse community with a couple of the most prominent doctors also receiving early infection. In Fig. 2b the infection is spread from the most prominent doctor, which was selected in Fig. 1b as the chosen spreader. Again the prominent nodes within this doctor’s community are amongst the earliest infected, with a selection of the most prominent nurses also being consistently infected within this initial time period. Since the initial disease spread is primarily contained within communities, distributing spreaders across these eigenvector-based communities can be an effective tactic, when looking to rapidly infect a network, as will be demonstrated in the following sections.

### Infection spread—real-world networks

**Fig. 3:**
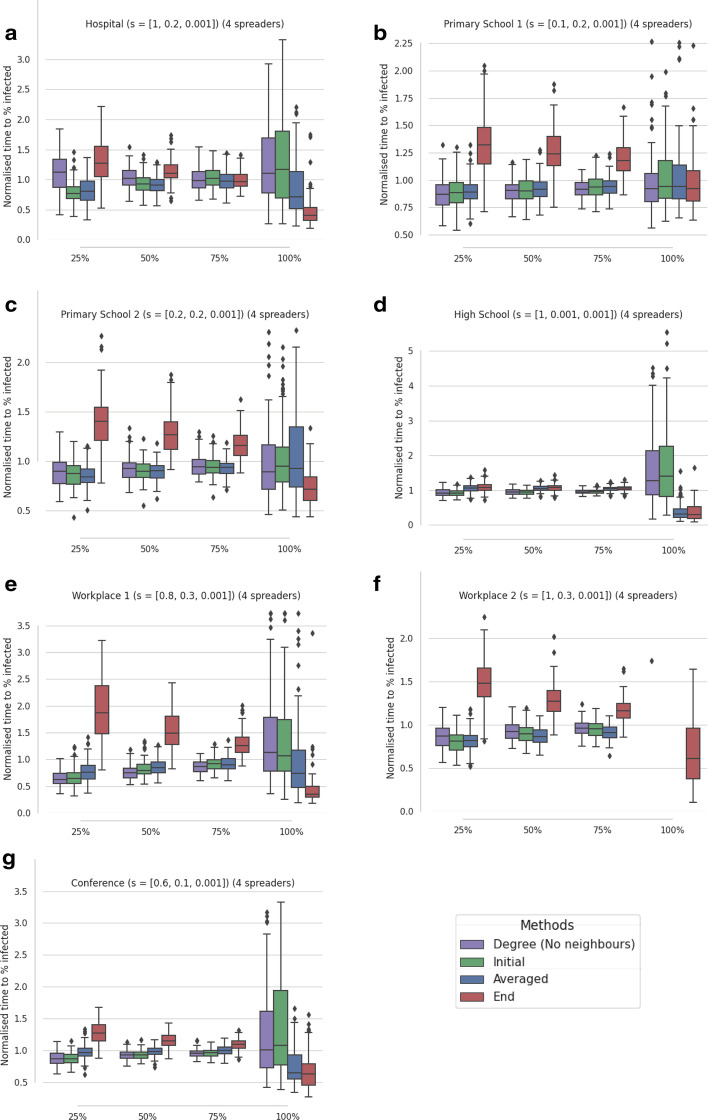
Results from SI simulations ($\tau = 1$) of real-world contact networks using four selected spreaders. The contact networks are, **a** a hospital ward (Vanhems et al. 2013), **b**, **c**, a primary school (Stehlé et al. 2011), **d** a high school (Salathé et al. 2010), **e**, **f** a workplace (Génois et al. 2015; Génois and Barrat 2018) and, **g** a conference (Génois and Barrat 2018). Times are recorded from 100 SI simulations when 25%, 50%, 75%, and 100% of network are infected and then normalised with the mean of all times recorded at each percentage. From left to right; times are detailed for the weighted degree (no neighbours) selection, then the eigenvector-based selections for initial rate (Initial), average performance (Averaged), and time to all infected (End). The y-axes of **a**, **c**, **f**, **g** are cropped to exclude extreme values

The ability to identify the most effective spreaders of disease is of most relevance when applied to real-world contact networks, such as those generated from data gathered using proximity sensors. In this section, the performance of spreader selection is evaluated in Fig. 3 for seven real-world contact networks. These contact networks include a hospital ward (Fig. 3a; $\text {N}=75$), a primary school (b, c $\text {N}=235$ and $\text {N}=238$), a high school (d $\text {N}=788$), a workplace (e, f; $\text {N}=92$ and $\text {N}=217$), and a conference (g; $\text {N}=403$).

The results in Fig. 3 highlight how the eigenvector-based selections can achieve three different measures of effectiveness, by varying the *s* value when creating the exponentially adjusted Laplacian matrix that represents the system. Thereby achieving fast times to 25% infection with *Initial*, consistently good performance with *Averaged*, or fast times to 100% infected with *End*. The *s* values selected are detailed above each plot in Fig. 3 for these three selections where ${s=[Initial, Averaged, End]}$.

The performance of weighted degree (no neighbours) appears to be most similar to the *Initial* selection, where there is only a clear difference in the time to 25% for Fig. 3a, f with *Initial* producing a faster infection rate. The *End* selection almost always produces the fastest time to 100% infection, but this can be seen to come at the cost of performance for all prior percentage intervals. The *End* selection also demonstrates that isolated nodes and communities appear to be common in human contact networks, as there is a very significant benefit to using this selection for the 100% infected times in Fig. 3a, c, d, e, f, g. The *Averaged* selection consistently positions itself between the times recorded for the *Initial* and *End* selections. In Fig. 3a, e, g this ensures a good performance at 100%, but without the large sacrifice in performance at lower percentage intervals.

These trends are not specific to the numbers of spreaders in the system, where results are detailed in the Additional file 1: Fig. S3 for various numbers of selected spreaders. These results also include comparisons with eigenvector centrality, betweenness centrality, and s-core (no neighbours). S-core (no neighbours) performs similarly to the weighted degree (no neighbours) metric, while eigenvector and betweenness centrality are frequently the least accurate metrics for selecting effective disease spreaders. Finally it is worth noting that for these examples, and those that follow, that varying $\tau$ does not result in changes to the relative performance of these methods and is not a focus of this paper for that reason.

### Infection spread—artificial networks

It is useful to investigate the consistency of spreader selection performance on networks with controllable topology. Proximity Graphs are used here to demonstrate that the performance of both the eigenvector-based and weighted degree (no neighbours) selections can vary with the topology. The definition of the proximity graph used here, referred to as proximity-nearest neighbour (P-NNR), is as follows: Distribute 100 points—representing network nodes—in a Euclidean plane with a uniform random distribution for the x and y coordinates between 0 and 1. Define a proximity threshold (d), and any two points separated by a Euclidean distance that is less than d are connected. The weights of all connections are then defined using a uniform random distribution between 0 and 1.

**Fig. 4:**
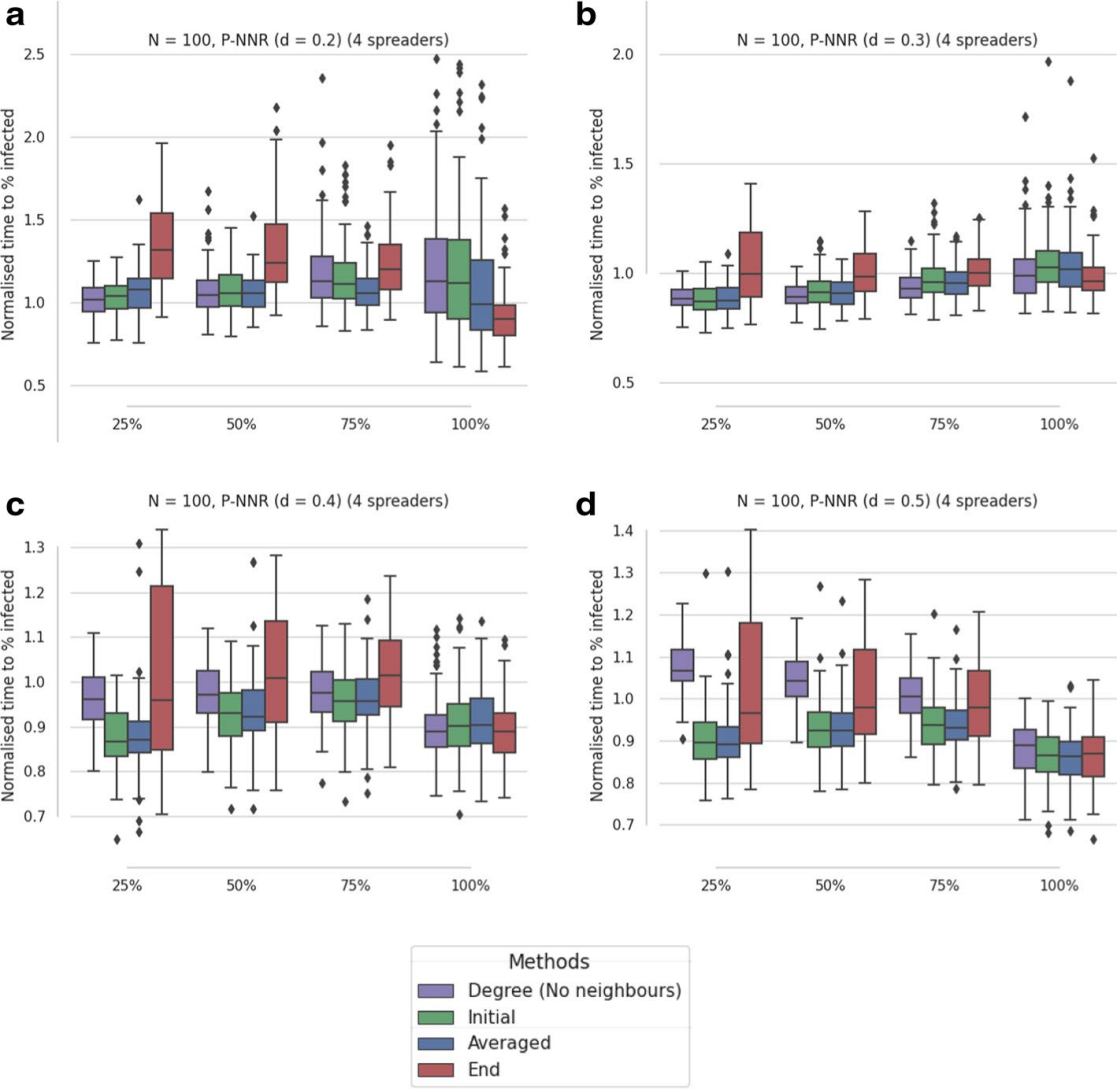
Results from SI simulations ($\tau = 1$) of artificial contact networks using four selected spreaders. The median times from 100 artificially generated proximity networks are reported, where each median was assessed from 100 SI simulations. The proximity networks (P-NNR) are defined by the Euclidean distance threshold (d). In **a** d = 0.2, **a** d = 0.3, **a** d = 0.4, and **a** d = 0.5 where connection weights are given a value between 0 and 1 according to a uniform random distribution. Times are recorded when 25%, 50%, 75% and 100% of network are infected then normalised using division by the mean of all times recorded at each percentage. From left to right; times are detailed for the *weighted degree (no neighbours)* selection, then the optimised selections for initial rate (*Initial*), average performance (*Averaged*), and time to all infected (*End*). The y-axes of **a**, **b** are cropped to exclude extreme values

In Fig. 4, 100 P-NNR graphs are investigated, with four different d values defining the topology construction, where each graph undergoes 100 SI simulations. While the eigenvector-based selections appear to perform similarly to the examples in Fig. 3, the $\text {d}=0.2$ and $\text {d}=0.3$ cases (Fig. 3a, b) require an alternative selection of *s* values. These results are achieved by employing a smaller range for the set *S* of possible values, namely $S=\{0.001,0.01,0.02,0.03,0.04,0.05,0.06,0.07,0.08,0.09,0.1\}$. The nominal range was defined between 0.001 and 1, but this produces a notably poorer performance in the $\text {d}=0.2$ and 0.3 cases for the *Initial* and *Averaged* selections. P-NNR topologies can produce strongly connected hubs, with the requirement for smaller *s* values likely necessary to reduce the influence of the most prominent community after the first chosen spreader’s removal and therefore enabling the selection of spreaders from less influential communities.

The weighted degree (no neighbours) selection also has a variable performance dependent on the d value. In particular, for $\text {d}\ge 0.4$ the weighted degree selection no longer produces a fast initial spread. This appears to be due to the no neighbours constraint, with larger d values creating more neighbours for each node. Therefore, there are fewer nodes that are not connected to a chosen spreader and hence available for selection. In fact, for $\text {d}=0.5$ the s-core and weighted degree selections are allowed to select any available nodes when there are no nodes remaining that are not connected to a chosen spreader.

### Ant pathogen response

The previous results emphasise the importance of community structure to disease spread in contact networks. By understanding the role of communities in effectively spreading disease, it is also possible to highlight effective network structures for preventing spread. In this section, the adaptation of ant colony contact networks in the presence of pathogen spreaders shall be presented, using the network eigenvectors, in a similar manner to Figs. 1 and 2.

**Fig. 5:**
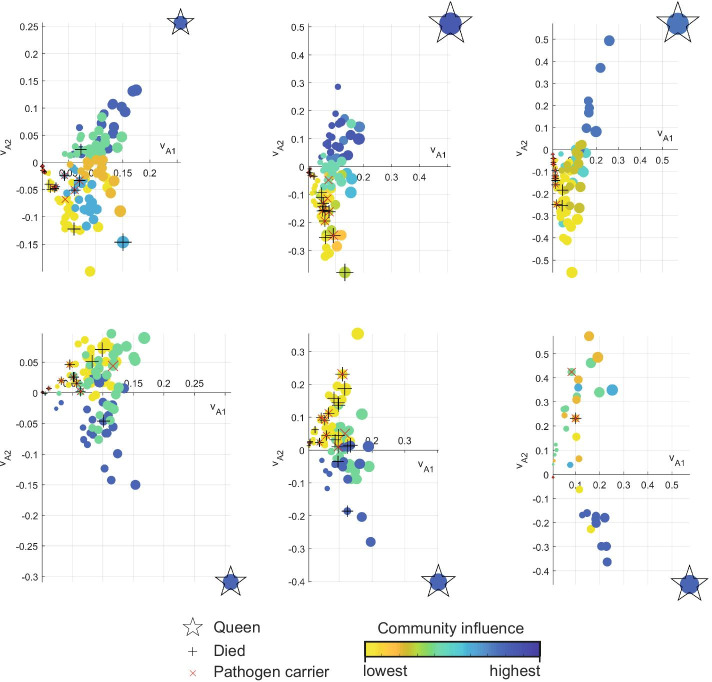
Community structure from ant colony contact network after pathogen introduction. CDI determined communities are detailed for contact networks of ant colonies infected with a pathogen during a survival experiment [see Stroeymeyt et al. 2018]. Node size is proportional to weighted degree, and colours denote community designation with each community’s influence ranked according to the magnitude of the largest entry of the first eigenvector ($\text {v}_{A1}$). There are markers indicating the queen ant, ants carrying pathogen at the start of the experiment and ants that died during the experiment. $\text {v}_{A1}$ and $\text {v}_{A2}$ are the two dominant eigenvectors of the adjacency matrix composed of pairwise contact durations

The experiment reported in Stroeymeyt et al. (2018) exposed a number of ants to a pathogen, monitored all ant contacts for a period of 9 days and recorded which ants died during that period. The separation of the queen ant from the pathogen carriers is noted by Stroeymeyt et al. (2018), and can also be seen clearly by looking at Fig. 5 where the network is embedded in a Euclidean space defined by the system eigenvectors with the communities of dynamical influence (CDI), described in the Communities of dynamical influence section, also detailed. Six of the eleven monitored colonies are presented in the figure. From these it can be seen that infected ants can still maintain relatively long durations of contact, as denoted by the size of each node’s marker that is proportional to weighted degree. There are examples of infected ants with minimal contacts during the experiment, but a more consistent sign of colony adaptation to the presence of pathogen is seen in the network structure. The communities are defined by CDI using the first three eigenvectors of the adjacency matrix, with Fig. 5 presenting two of these three dimensions. In Fig. 5, the queen’s community is either free from pathogen carriers or pathogen carriers are at the origin of the eigenvector defined Euclidean space, which indicates that they have very limited contact with other ants. In many of the examples, no ants in the queen’s community die during this survival experiment, indicating that they are unlikely to have contracted significant quantities of the pathogen. Furthermore, the relative influence of communities are indicated in Fig. 5, based on the largest first eigenvector entry of the adjacency matrix (eigenvector centrality) from each community. Pathogen carrying ants are commonly located in the least influential communities, as seen for 8 out of the 11 colonies investigated, where they have less ability to infect the rest of the network. Whereas pathogen carriers were only present in the most influential community in 2 out of the 11 colonies. In both of these cases, the pathogen carriers were closer to the origin of the Euclidean frame than the majority of their community’s members, indicating their lack of influence in this most influential community. All 11 ant colonies are presented in the Additional file 1, including both the $\text {v}_{A1}$ and $\text {v}_{A2}$ (Fig. S4) and $\text {v}_{A2}$ and $\text {v}_{A3}$ (Fig. S5) perspectives.

For the survival experiments in Stroeymeyt et al. (2018), the pathogen was given exclusively to forager ants which are those most likely to pick it up when venturing outside of the nest. It is, therefore, interesting to note that this separation of forager communities from the queen is detected even in colonies that were monitored when none were infected and provides an example of effective network topology for mitigating epidemic spread. As forager isolation after infection, noted by Stroeymeyt et al. (2018), can be achieved without significant reorganisation of the colony’s contact network.

Finally, Fig. 5 presents the eigenvectors of the adjacency composed of contact duration, rather than the exponentially adjusted Laplacian, as the probability of pathogen spread in ants differs from that of human disease spread. For ants carrying pathogens, Stroeymeyt et al. (2018) notes that the probability also depends on the quantity of pathogen spores an ant is carrying.

## Discussion

The manipulation of the Laplacian matrix to better represent the dynamics of disease spread highlights a potential pitfall for centrality measures where the best performing measure can be chosen without a clear justification for why it should be effective [see Brandes (2020) for further discussion on the appropriate use of centrality measures]. In this case, it is logical that weighted degree should perform well when identifying effective spreaders, but given the clear relationship between disease spread and random walk assessments it is less reasonable that it should significantly and consistently outperform an eigenvector-based assessment. This paper has demonstrated that there is an issue with analysing the contact network composed of contact durations, when what is of primary concern is the probability of disease transmission. We have attempted to provide a better representation of a disease transmission network, by introducing the exponentially adjusted Laplacian. In doing so, we have highlighted why weighted degree selections perform well in the specific application of disease spread; this is not a new observation as De Arruda et al. (2014) notes that selection metrics differ depending on the underlying dynamics. But here we go further by illuminating why these dynamics essentially alter the balance between the probabilities of spreading and receiving disease, which amplify the influence of high degree nodes.

The three spreader selections *Initial*, *Averaged*, and *End* take advantage of understanding the trade-off between the most influential nodes in terms of immediate neighbours (those with multiple, high weight, connections) and the nodes that are only locally influential but are part of isolated communities. An optimisation is required to make these selections, but the results show that reducing the magnitude of weights in the adjacency matrix, before creating the exponentially adjusted Laplacian, changes selections from *Initial*, to *Averaged*, and then finally produces an *End* selection. This occurs because the scaling affects the ratio between incoming connection weights (relative probabilities of spreading infection) and the magnitude of the diagonal matrix entry (the relative probability of receiving infection).

The *Initial* and *Averaged* selections are of obvious interest to disease spread, both in terms of targets for testing—where effective spreaders would be the most damaging to go unnoticed - and potentially as candidates for vaccination. However, testing and vaccination optimisation are notably different problems to the detection of effective spreaders, so for example there is no guarantee that the most effective spreaders are the most effective to vaccinate. Also the spread of disease on susceptible-infected-susceptible (SIS) simulations has been shown by Nadini et al. (2018) to differ from the susceptible-infected-recovered (SIR) models of infection spread, so these results may not translate exactly to real-world implementation.

*End* selections may be of limited interest in preventing disease spread in humans, where the focus usually centres on preventing rapid initial spread of infection. But instead there may be applications in pursuits such as the sterilisation of mosquitoes through the intentional spread of genes as described in Callaway (2015), where the goal is to spread engineered genes throughout the entire populace.

The link between eigenvector-based spreader selection—that incorporates connection removal—and the communities defined by CDI is explored in the hospital ward contact network (Fig. 1), where weighted degree and also eigenvector centrality would fail to recognise the importance of targeting the less influential community comprised of doctors. This raises the question of why community detection is not part of Algorithm 1 for identifying spreaders, but as can be seen in Fig. 1 and as demonstrated by Clark et al. (2016) the removal of node connections is a method for revealing network communities. In the context of disease spread, it also provides a more accurate representation of the system after node infection, given that infected nodes cannot be influenced (infected) by others.

Another observation from the hospital ward network (Fig. 1) is its division into communities dominated by influential nurses and doctors, with patients presenting as far less effective spreaders. Focused testing in health care facilities was a recommendation from the World Health Organisation for combating the COVID-19 epidemic [see World Health Organization and others (2020)]. Given the insights found on a hospital ward network of 75 people, a similar application could be envisioned for key hospital wards where proximity sensors are deployed and the insights of network analysis used to inform testing and interventions in response to an infection outbreak.

The majority of this paper concerns the detection of effective spreaders using the system’s eigenvectors, when accounting for the exponential relationship between contact duration and risk of transmission. For the ant colony examples, the relationship between contact duration and risk of transmission depends on the quantity of pathogen spores, see Stroeymeyt et al. (2018). Modelling this ant pathogen transmission risk is beyond the scope of the paper, therefore the eigenvectors are assessed using an adjacency matrix of contact durations. This emphasises that contact duration analysis can still provide insights into the spreading dynamics of a system and, in this case, the effective adaptation of ant colony structure. However, as has been shown throughout this paper, the analysis of the ant colonies using a matrix representation that captures the risk of transmission, and not just contact duration, would be more accurate and possibly more insightful.

## Conclusions

Network structure influences the location of the most effective spreaders of disease; evidenced by ant colony networks and the effectiveness of eigenvector-based identification of effective spreaders, on both simulated and real-world contact networks. The system eigenvectors capture the influence of disease spreaders, when the network dynamics are accurately represented. We show that disease spread dynamics can be approximated by adjusting the construction of the Laplacian matrix to capture the non-linear relationship between contact time and the probability of disease spread. By representing the dynamics in this way, the success of degree based metrics—in identifying effective spreaders of disease—is shown to be due, in part, to the non-linear dynamics rewarding high degree nodes with greater influence. The concept of an effective spreader is shown to be ill-defined, where—for the newly introduced exponentially adjusted Laplacian—altering the ratio of incoming connection weight versus the magnitude of the diagonal entry creates a trichotomy on the concept of effective spreading. A spreader selection can be identified that can outperform the benchmark metric of weighted degree (applying a no neighbour constraint) in terms of the initial rate of infection, the average rate of infection, or the time to full infection of the network.

## Supplementary Information

**Additional file 1**. Provides additional results, including performance comparison of other benchmark spreader selections (Fig. S1 and S2), the effect of varying the number of spreaders selected (Fig. S3), and results for all 11 ant colonies from the survival experiment (Fig. S4 and S5).

## Data Availability

The ant survival experiment datasets analysed during the current study are available in the Zenodo repository, https://doi.org/10.5281/zenodo.1322669. The contact network datasets analysed during the current study are available in the SocioPatterns repository, http://www.sociopatterns.org/datasets/.

## Abbreviations

CDI: Communities of dynamical influence
P-NNR: Proximity nearest neighbour
SI: Susceptible-infected
SIS: Susceptible-infected-susceptible
SIR: Susceptible-infected-recovered
