# Supplementary material for: Identification of effective spreaders in contact networks using dynamical influence
Source: Appl Netw Sci. 2021 Jan 19;6(1):5. doi: 10.1007/s41109-021-00351-0 (PMC7814176; doi:10.1007/s41109-021-00351-0)
Supplement: Supplementary file 1 — Additional file 1. Provides additional results, including performance comparison of other benchmark spreader selections (Fig. S1 and S2), the effect of varying the number of spreaders selected (Fig. S3), and results for all 11 ant colonies from the survival experiment (Fig. S4 and S5). [file 41109_2021_351_MOESM1_ESM.pdf]

RESEARCH

Supplemental material: Identification of effective spreaders in contact networks using dynamical influence

Ruaridh A Clark* and Malcolm Macdonald

*Correspondence: ruaridh.clark@strath.ac.uk
Department of Electronic and Electrical Engineering, University of Strathclyde, George Street, Glasgow, UK
Full list of author information is available at the end of the article

S1 Spreader selection benchmarks

The results from comparisons with other benchmarks – including eigenvector centrality, s-core strategy (a weighted degree version of k-core/k-shell introduced by Eidsaa and Almaas (2013)), and betweenness centrality – are shown here alongside the weighted degree (no neighbours) and the three eigenvector-based selections using the exponentially adjusted Laplacian matrix. The no neighbour constraint, whereby nodes cannot be chosen as spreaders if they are directly connected to an already selected node, is also applied here for the s-core strategy. The results from the main paper are duplicated for these seven selections and presented in Fig. S1 for the real-world contact networks and in Fig. S2 for the proximity network (P-NNR).

S2 Number of spreaders

The paper focuses on selecting four spreaders, but the performance of the three eigenvector-based selections are shown in Fig. S3 to be similar when selecting 2, 4, 6, or 8 spreaders on the hospital ward contact network. It is interesting to note that there is still a clear trade-off between selections for fast initial response and full network infection when only selecting two spreaders, see **a**. Also note that the weighted degree (no neighbours) method cannot produce selections of more than 9 spreaders without violating the no neighbour constraint as no nodes are left without a direct connection to a selected spreader.

S3 Ant colony results

The following Fig. S4 and S5 presents all 11 ant colonies from the survival experiment conducted by Stroeymeyt et al. (2018). The colony IDs, defined in Stroeymeyt et al. (2018), are as follows; **a**: 004, **b**: 007, **c**: 008, **d**: 010, **e**: 011, **f**: 014, **g**: 025, **h**: 032, **i**: 045, **j**: 049, and **k**: 055. In Fig. S4, the axes are the first two eigenvectors of the adjacency matrix (v_{A1} and v_{A2}). In Fig. S5, the axes are the second and third eigenvectors of the adjacency matrix (v_{A2} and v_{A3}). Communities are defined as communities of dynamical influence, see Clark et al. (2019). The queen ant is marked in each plot, except in **l** where the data was not available but the queen is likely to be the high weighted degree node at the head of the most influential community.

In the paper, we report that the queen is frequently separated from the infected nodes where they are often on opposite sides of the v_{A2} axis. In Fig. S4 **a**, **f**, **i**, and

k this is not the case with some of the infected nodes on the same side. However,

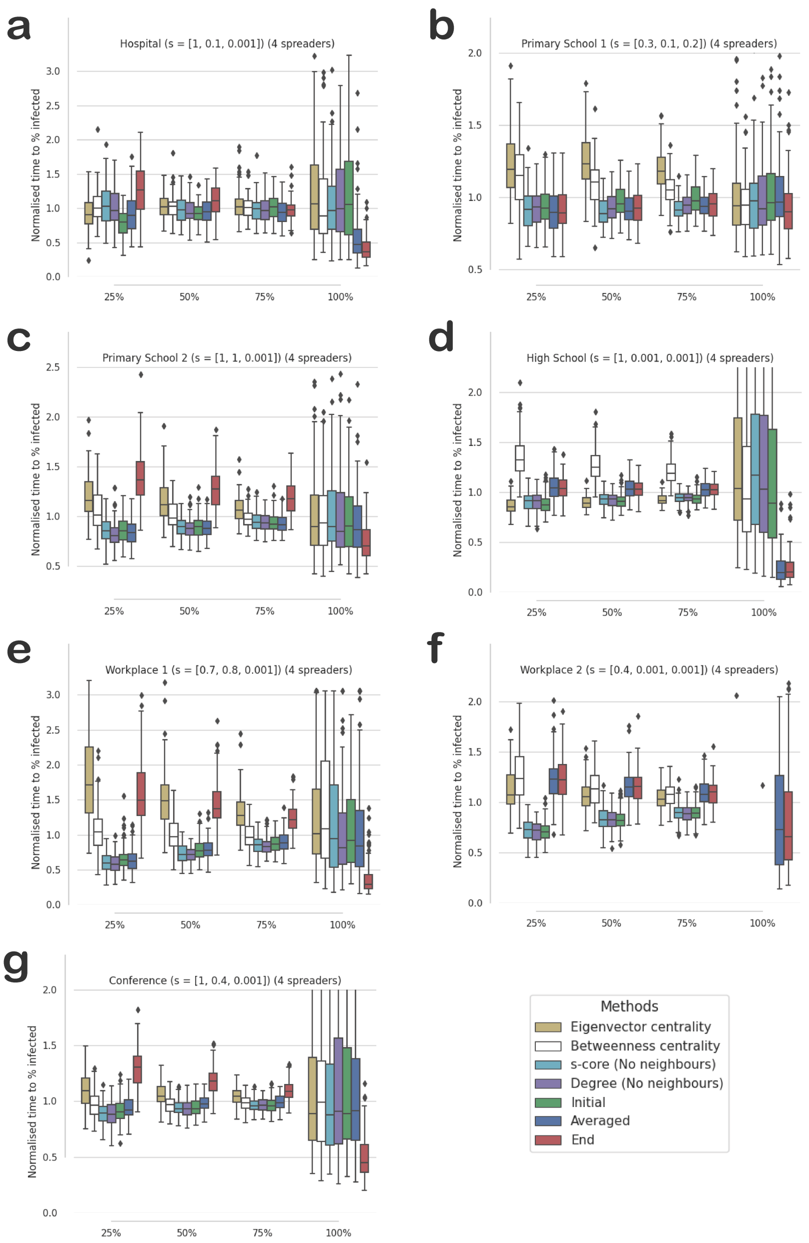

Figure S1 Results from SI simulations ($\tau = 1$) of real-world contact networks using four selected spreaders. The contact networks are, **a**, a hospital ward (Vanhems et al. (2013)), **b** & **c**, a primary school (Stehlé et al. (2011)), **d**, a high school (Salathé et al. (2010)), **e** & **f**, a workplace (Génois and Barrat (2018); Génois et al. (2015)) and, **g**, a conference (Génois and Barrat (2018)). Times are recorded from 100 SI simulations when 25%, 50%, 75%, and 100% of network are infected and then normalised with the mean of all times recorded at each percentage. From left to right; times are detailed for the weighted degree (no neighbours) selection, then the eigenvector-based selections for initial rate (Initial), average performance (Averaged), and time to all infected (End). The y-axes of **a**, **c**, **f** and **g** are cropped to exclude extreme values.

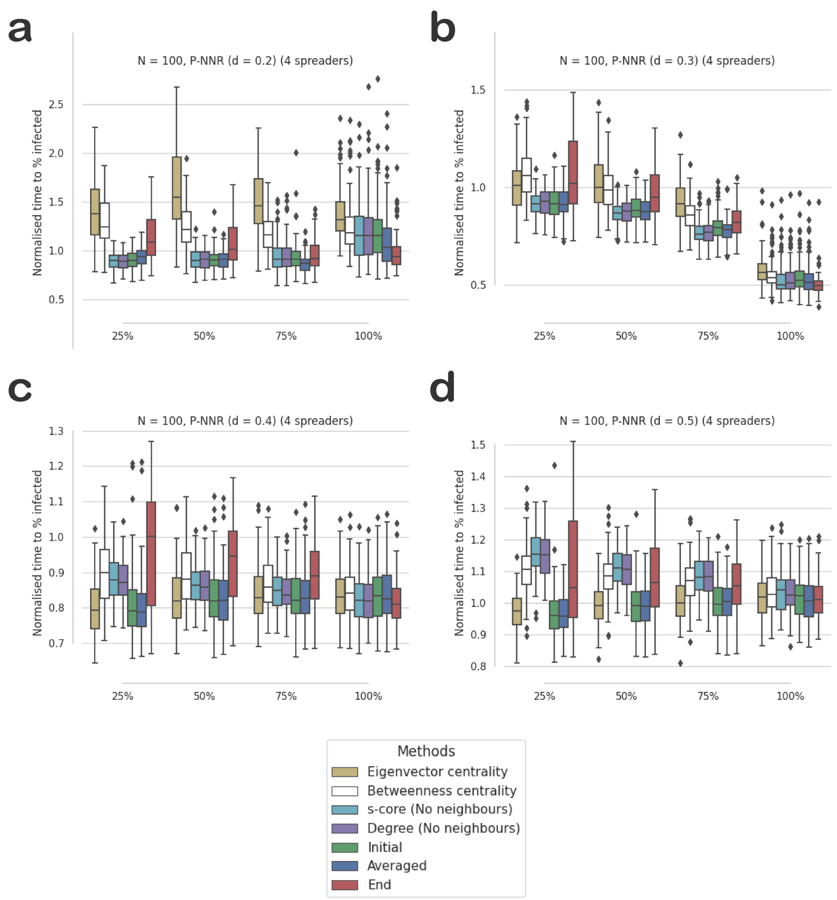

Figure S2 Results from SI simulations ($\tau = 1$) of artificial contact networks using four selected spreaders. The median times from 100 artificially generated proximity networks are reported, where each median was assessed from 100 SI simulations. The proximity networks (P-NNR) are defined by the Euclidean distance threshold (d). In **a** d=0.2, **a** d=0.25, **a** d=0.3, and **a** d=0.35 where connection weights are given a value between 0 and 1 according to a uniform random distribution. Times are recorded when 25%, 50%, 75% and 100% of network are infected then normalised using division by the mean of all times recorded at each percentage. From left to right; times are detailed for eigenvector centrality, betweenness centrality, s-core (no neighbours), *weighted degree (no neighbours)* selection, then the optimised selections for initial rate (*Initial*), average performance (*Averaged*), and time to all infected (*End*). The y-axes of **a** and **b** are cropped to exclude extreme values.

Fig. S5 shows that in almost every colony, except **k**, the queen has no infected nodes in the same quadrant when the axes are v_{A2} and v_{A3}.

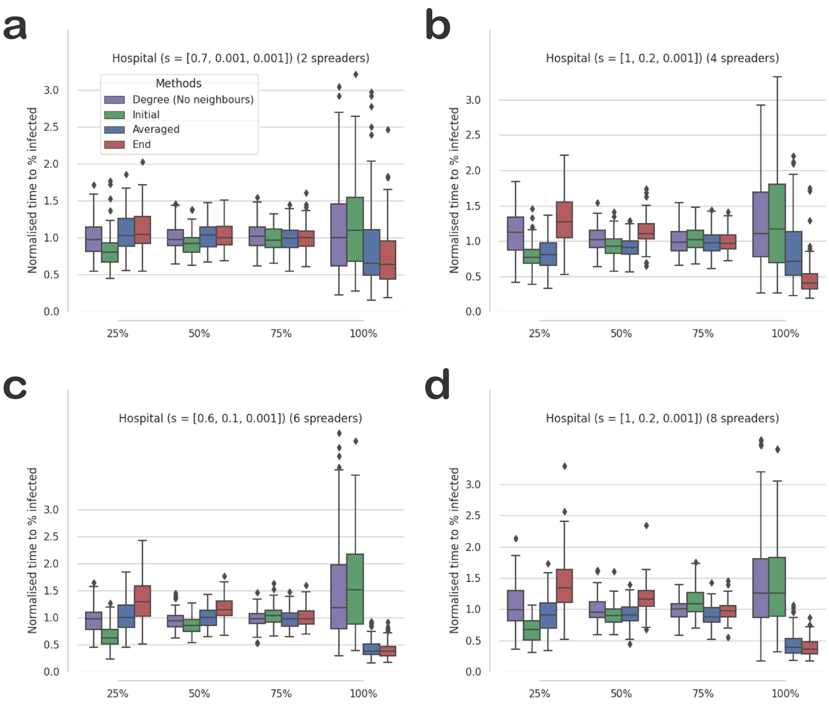

Figure S3 Results from SI simulations ($\tau = 1$) of a hospital ward contact network using varying numbers of selected spreaders. Times are recorded from 100 simulations of disease spread on a hospital ward contact network (see Vanhems et al. (2013)) with $\mathbf{a}$ 2 spreaders, $\mathbf{b}$ 4 spreaders, $\mathbf{c}$ 6 spreaders and $\mathbf{d}$ 8 spreaders. The times are reported when 25%, 50%, 75%, and 100% of network are infected and then normalised using division by the mean of all times recorded at each percentage. From left to right; times are detailed for eigenvector centrality, betweenness centrality, s-core (no neighbours), weighted degree (no neighbours) selection, then the optimised selections for initial rate (Initial), average performance (Averaged), and time to all infected (End). The y-axes are cropped in each plot to exclude extreme values.

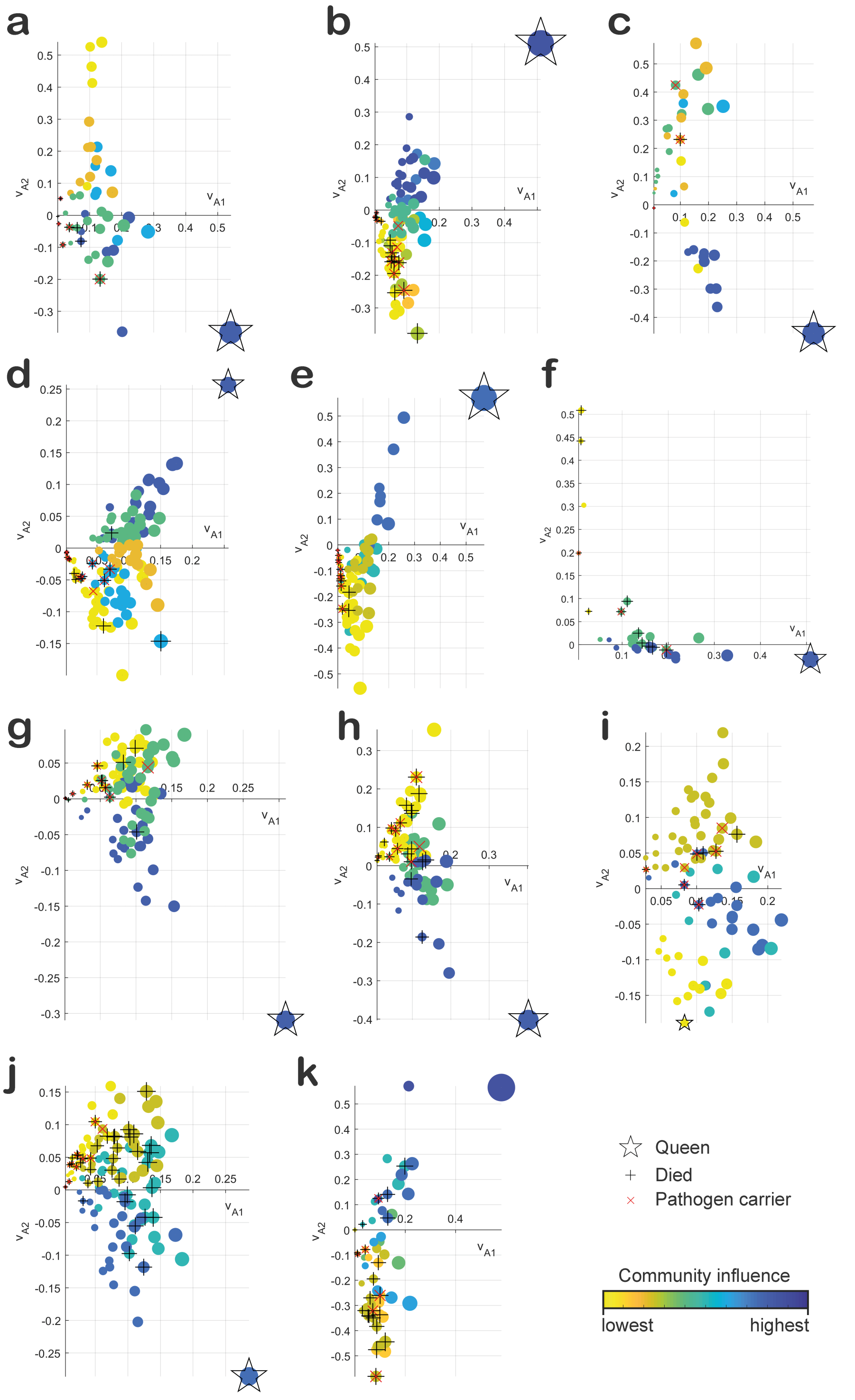

Figure S4 Community structure from ant colony contact network after pathogen introduction. CDI determined communities are detailed for contact networks of ant colonies infected with a pathogen during a survival experiment (see Stroeymeyt et al. (2018)). Node size is proportional to weighted degree, and colours denote community designation with each community's influence ranked according to the magnitude of the largest entry of the first eigenvector (v_{A1}). There are markers indicating the queen ant, ants carrying pathogen at the start of the experiment and ants that died during the experiment. v_{A1} and v_{A2} are the two dominant eigenvectors of the adjacency matrix composed of pairwise contact durations.

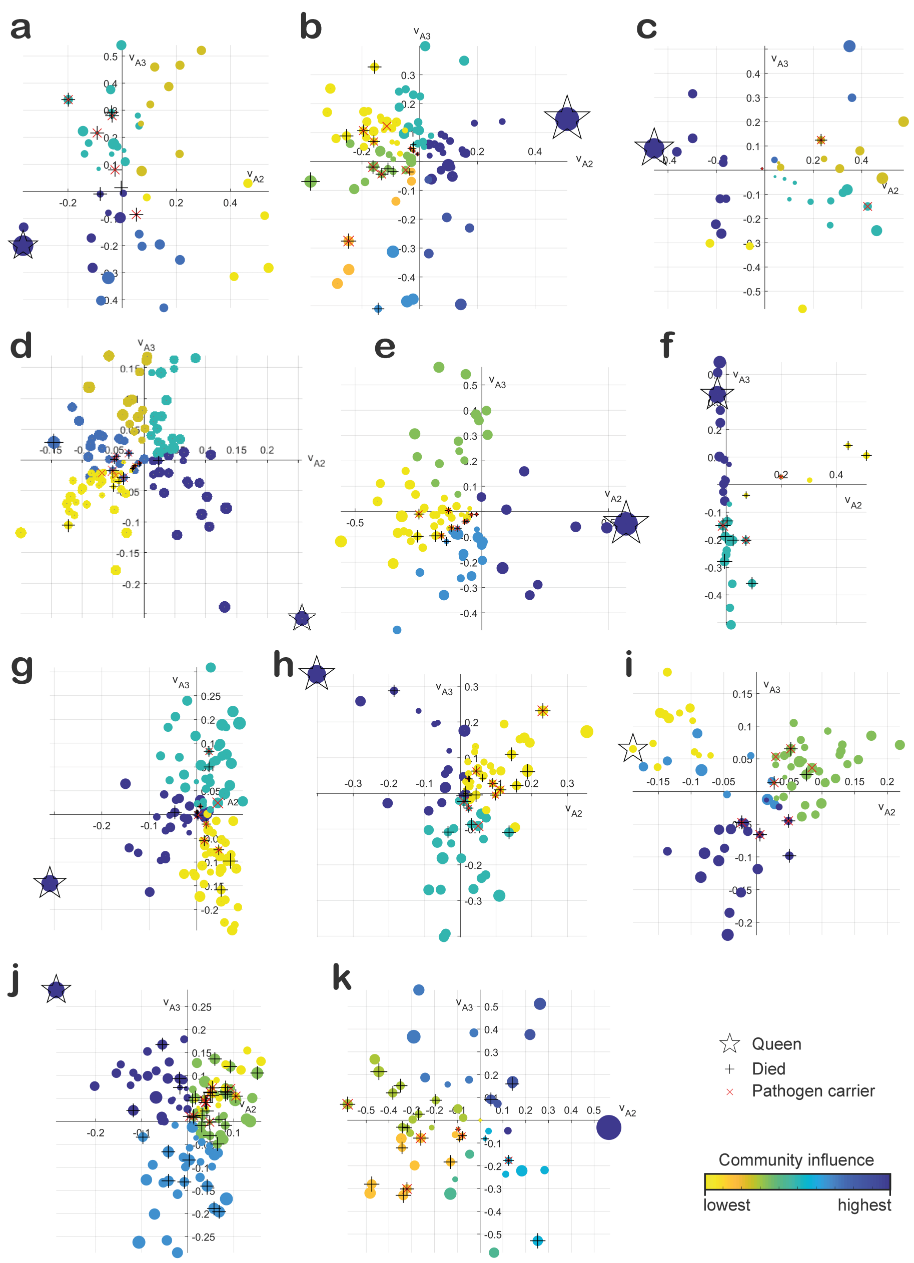

Figure S5 Community structure from ant colony contact network after pathogen introduction. CDI determined communities are detailed for contact networks of ant colonies infected with a pathogen during a survival experiment (see Stroeymeyt et al. (2018)). Node size is proportional to weighted degree, and colours denote community designation with each community's influence ranked according to the magnitude of the largest entry of the first eigenvector (v_{A1}). There are markers indicating the queen ant, ants carrying pathogen at the start of the experiment and ants that died during the experiment. v_{A2} and v_{A3} are the second and third eigenvectors of the adjacency matrix composed of pairwise contact durations.

References

Clark, R., Punzo, G., Macdonald, M.: Network communities of dynamical influence. Scientific reports **9**(1), 1–13 (2019)

Eidsaa, M., Almaas, E.: S-core network decomposition: A generalization of k-core analysis to weighted networks. Physical Review E **88**(6), 062819 (2013)

Génois, M., Barrat, A.: Can co-location be used as a proxy for face-to-face contacts? EPJ Data Science **7**(1), 11 (2018). doi:10.1140/epjds/s13688-018-0140-1

Génois, M., Vestergaard, C.L., Fournet, J., Panisson, A., Bonmarin, I., Barrat, A.: Data on face-to-face contacts in an office building suggest a low-cost vaccination strategy based on community linkers. Network Science **3**, 326–347 (2015). doi:10.1017/nws.2015.10

Salathé, M., Kazandjieva, M., Lee, J.W., Levis, P., Feldman, M.W., Jones, J.H.: A high-resolution human contact network for infectious disease transmission. Proceedings of the National Academy of Sciences **107**(51), 22020–22025 (2010)

Stehlé, J., Voirin, N., Barrat, A., Cattuto, C., Isella, L., Pinton, J.-F., Quaggiotto, M., Van den Broeck, W., Régis, C., Lina, B., *et al.*: High-resolution measurements of face-to-face contact patterns in a primary school. PloS one **6**(8), 23176 (2011)

Stroeymeyt, N., Grasse, A.V., Crespi, A., Mersch, D.P., Cremer, S., Keller, L.: Social network plasticity decreases disease transmission in a eusocial insect. Science **362**(6417), 941–945 (2018)

Vanhems, P., Barrat, A., Cattuto, C., Pinton, J.-F., Khanafer, N., Régis, C., Kim, B.-a., Comte, B., Voirin, N.: Estimating potential infection transmission routes in hospital wards using wearable proximity sensors. PloS one **8**(9), 73970 (2013)
